# Morphology and morphometry of the transverse foramina of cervical vertebrae in an adult Kenyan population: a radiological study

**DOI:** 10.4314/ahs.v24i2.38

**Published:** 2024-06

**Authors:** Khulud Nurani, Pamela M Idenya, James Kigera, Philip M Mwachaka

**Affiliations:** Department of Human Anatomy, School of Medicine, College of Health Sciences, University of Nairobi. P.O.Box 30197-00100, Nairobi, Kenya

**Keywords:** Morphology and morphometry, transverse foramina, cervical vertebrae, in Kenyan Population, a radiological study

## Abstract

**Background:**

Transverse foramina are canals in cervical transverse processes transmitting the vertebral vessels and the accompanying sympathetic plexus. These foramina exhibit side, sex and population specific variations such as those of size, shape and number. Knowledge of these variations is important for cervical surgical procedures and prediction of vertebral artery variations.

**Objective:**

To describe the morphology and morphometry of cervical transverse foramina in an adult Kenyan population.

**Methods:**

Ninety-four neck CT scan images of 2 mm slice thickness in axial view were used to assess presence, number, completeness and shape of transverse foramina. Antero-posterior and transverse lengths were measured using NeusoftTM software. Paired and independent t-tests were used to compare morphometric parameters for side and sex respectively. One-way ANOVA was used to determine differences in foramina down the cervical spine. A p-value of ≤ 0.05 was considered significant.

**Results:**

Transverse foramina had a prevalence of 98.78% with 3.64% being duplicated. They were identified as type 1 (circular), type 2 (elongated antero-posteriorly), type 3 (elongated transversely), type 4 (oblique left-right elongation) and type 5 (oblique right-left elongation) in 69.62%, 3.62%, 13.38%, 7.23% and 6.15% respectively. 0.46% foramina were incomplete and 3.19% constricted. Diameters on the right were larger than left. C1 foramina were the largest and C7 smallest. The diameters decreased from C2 to C3 then increased to C6.

**Conclusion:**

Transverse foramina display side and level dependent variations. This is of clinical importance to spine surgeons to prevent intraoperative damage of vertebral vessels when operating in the cervical region.

## Introduction

Transverse foramina are canals in the transverse processes of the cervical vertebrae that transmit the vertebral artery (VA), vertebral vein and accompanying sympathetic plexus 1. The transverse foramen (TF) of C6 provides an exit to the vertebral vein and an entrance to the vertebral artery and the surrounding nervous plexus, whereas the TF of C7 contains accessory vessels and nerve branches^2^. Variations in foraminal shape, number and dimension have been reported and noted to exhibit population, sex and side differences 3–5. These variations are significant due to their impact on the contents they transmit^3^. Narrow and spiculated foramina may predispose foraminal contents to compression. Compression of the VA may cause vertigo by impeding vertebrobasilar and subsequent labyrinthine circulation^6^, cervical myelopathy by impeding spinal circulation and Bow Hunter's stroke during head rotation by impeding cephalic circulation 5,7 Compression of the sympathetic plexus may cause cervicogenic syndromes and spasms in the vertebrobasilar arteries which can compromise labyrinthine circulation and cause hearing difficulties 8. Moreover, spicules of incomplete septation between the foramina may pierce the neurovascular structures resulting in vascular insufficiency or persisting pain 9.

There has been an increase in cervical spine pathologies^10^,^11^ and correspondingly, an increase in cervical vertebral surgical procedures which require extensive knowledge of the anatomy of the cervical vertebrae. Surgeries involving complex procedures like corpectomy, instrumentation, and treatment for neoplasms and traumas, have a high incidence of intraoperative VA injury which is further heightened during variations 12,13.

Knowledge of the morphology and morphometry of cervical TF is thus vital for surgical procedures such as cervical pedicle screw insertion, paravertebral foramen screw insertion, anterior and lateral decompression, cervical discectomy and fusion, cervical disk replacement and posterior cervical lamino-foraminotomy 14. Variations of the foramina are important to neurosurgeons in determining the etiology of vertebrobasilar insufficiency and vascular variations in the cervical region. They are also important to surgeons to avoid iatrogenic injury to the traversing neurovasculature during posterior and lateral approaches to the cervical spine 10. This study thus sought to describe the morphological and morphometric variations of cervical TF in an adult Kenyan population using computed tomography imaging.

## Materials and methods

This study was of descriptive cross-sectional design. Computed tomography (CT) scans of cervical vertebrae from adult Kenyan patients were obtained and assessed in the Radiology department, Kenyatta National Hospital. The study subjects were patients who had neck CT scans performed on them at Kenyatta National Hospital. OpenEpi online software was used to determine the number of CT scans that were used for the study. Using a power of 80%, a 95% confidence interval and the difference in mean of both sides expected to be clinically significant as 0.54 mm 5, the minimum sample size was calculated as 92 CT scans of the neck. Ninety-four CT scans of adult patients of both sexes were analyzed. CT scans were taken from individuals who had received examinations for reasons unrelated to this study. CT scans of participants with cervical vertebrae fractures, tumors or abnormalities such as hemivertebra, were excluded. Consecutive sampling was employed. Available CT scans of the neck region in the database that fit the selection criteria were used.

The study was approved by the Ethics and Research Committee. Subjects' information was kept strictly confidential. For consistency, standardization methods were used for scan view, scale, and horizontal reference plane. Datasheets were kept in password-protected folders. Data was uploaded from the databases to SPSS (version 28.0, Chicago, Illinois).

Cervical CT scans, extending from the base of the skull to the thoracic inlet, were studied using bone window imaging at a slice thickness of 2 mm. To achieve symmetry, CT scans were aligned in 3D- coronal, axial, and sagittal views. On the sagittal view, C2 dens was employed as reference for cervical numbering. The presence, number and completeness of the foramina was noted. Foramina were classified into five categories based on their shape using the criteria by Taitz et al. (1978). Anteroposterior and transverse diameters of TF were obtained in the axial view using an inbuilt ruler in the evaluation software NeusoftTM.

Data was checked for normalcy using Kolmogorov-Smirnov and Shapiro-Wilk tests and expressed as mean±standard deviation. An independent t-test was used for inter-individual comparisons of the morphometric parameters between males and females. A paired t–test was used for intra-individual comparison of parameters on each side. One-way ANOVA was used to determine morphometric differences down the cervical spine. A Tukey Post Hoc multiple comparisons test was then used to establish levels of greatest difference. A p-value of ≤ 0.05 was considered significant at a confidence interval of 95%.

## Results

Examination and analysis of 1316 TF from the 94 CT scan images was done for morphologic and morphometric variations. Sixty-one (64.9%) of the CT scans were from males and thirty- three (35.1%) were from females. Data was characterized as normal after passing it through Kolmogorov-Smirnov and Shapiro-Wilk normalcy tests. Morphology

**Figure 1:**
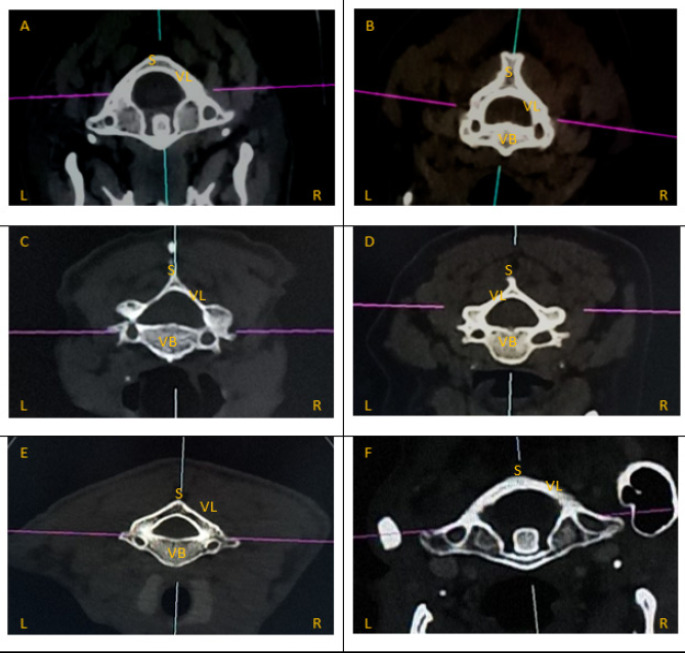
Types of TF based on Shape Axial view of Cervical CT scans illustrating different Foraminal Shapes **R-** Right Side; **L-** Left Side; **VB-** Vertebral Body; **L-** Lamina; **S**-Spine **FIGURE 1A-** Atlas displaying Type 1/circular TF on right and left sides **FIGURE 1B-** Axis displaying Type 2/antero-posteriorly elongated TF on right and left sides **FIGURE 1C-** C3 displaying Type 1/circular TF on the right and Type 2/antero-posteriorly elongated on the left **FIGURE 1D-** C5 displaying Type 3/transversely elongated TF on right and left sides **FIGURE 1E-** C7 displaying a Type 4 (oblique, elongated from left to right) TF on the right and Type 5 (oblique from right to left) on the left **FIGURE 1F-** Atlas displaying Type 5 (oblique from right to left) TF on right and Type 4 (oblique, elongated from left to right) on the left

Of the TF, 16 TF (1.22%) were absent. Of these, 15 (93.75%) were absent at C7 and 1 (6.25%) at C1. The most common shape was type 1 seen in 69.62% (905/1300 present TF), followed by type 3 in 13.38% (174/1300 present TF), type 4 in 7.23% (94/1300 present TF), type 5 in 6.15% (80/1300 present TF) and type 2 in 3.62% (47/1300 present TF). Figure 1 illustrates the various foraminal shapes encountered.

Type 1 was the most common foraminal shape (69.62%) and type 2 the least (3.62%) in both sexes. Types of TF were cross tabulated with sex. Chi-square test results indicated no statistically significant sex differences in shapes of TF (p=0.927).

In addition, types of TF were cross tabulated with right and left sides. Type 1, 2 and 4 were more frequent on the right side and type 3 and 5 more frequent on the left. Type 1 on the left were 437 versus 468 on the right, type 2 23 on the left versus 24 on the left, type 3 107 on the left versus 67 on the right, type 4 10 on the left versus 84 on the right and type 5 70 on the left and 10 on the right. Chi square test results indicated s/span>tatistically significant side differences in shapes of TF between the right and left side (p < 0.001).

Also, types of TF were cross tabulated with cervical vertebral levels. Chi square test results indicated statistically significant level differences in shapes of TF down the cervical spine (p < 0.001). Type 1 and 3 were more common at C1, C3, C4 and C5, Type 2 was more common at C2 and Types 4 and 5 were more common at C7.

### Duplication

**Figure 2:**
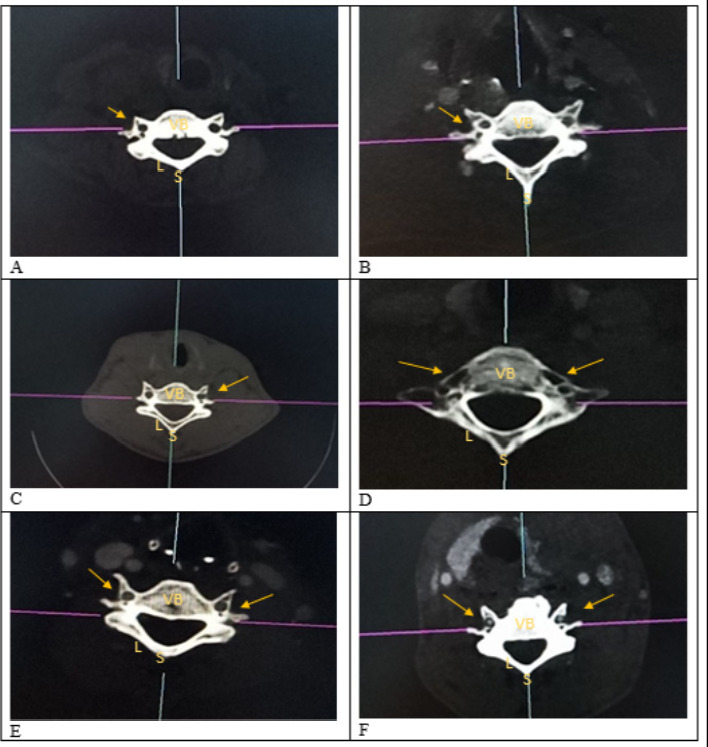
Varieties of Accessory TF Axial view of Cervical CT scans illustrating Accessory Foramina **VB-** vertebral body; **L-** vertebral lamina; **S**-spine **FIGURE 2A -** C6 displaying right unilateral complete double foramina (yellow arrows) **FIGURE 2B -** C6 displaying right unilateral complete double foramina (yellow arrows) **FIGURE 2C** - C4 displaying left unilateral complete double foramina (yellow arrows) **FIGURE 2D-** C7 displaying right triplication and left complete double foramina (yellow arrows) **FIGURE 2E-** C6 displaying bilateral complete double foramina (yellow arrows) **FIGURE 2F** –C6 displaying bilateral incomplete double foramina (yellow arrows)

Of the 658 vertebrae studied, 24 had duplicated TF (3.64%). Four duplications were at C1 vertebral level (16.7%), 1 at C4 (4.2%), 2 at C5 (8.3%), 16 at C6 (66.7%) and 1 at C7 (4.2%). The accessory foramina were noted as being smaller than parent foramina. Varieties of accessory foramina are illustrated below (Figure 2).

### Completeness

**Figure 3:**
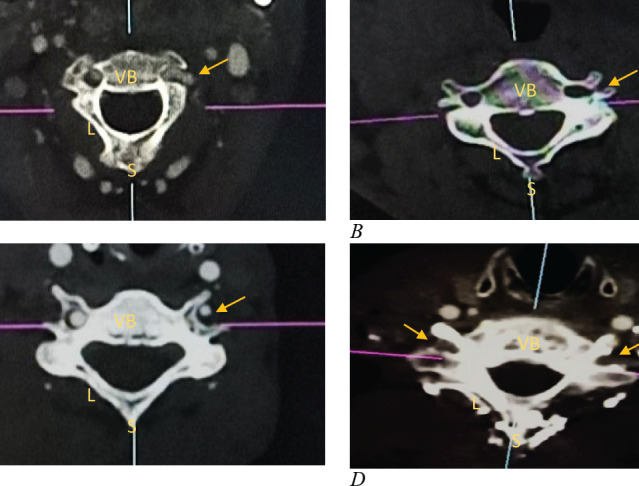
Illustration of Incomplete Transverse Foramina

Of the TF studied, six were noted as incomplete. This is illustrated below (Figure 3).

### Constricted and Spiculated Foramina

**Figure 4:**
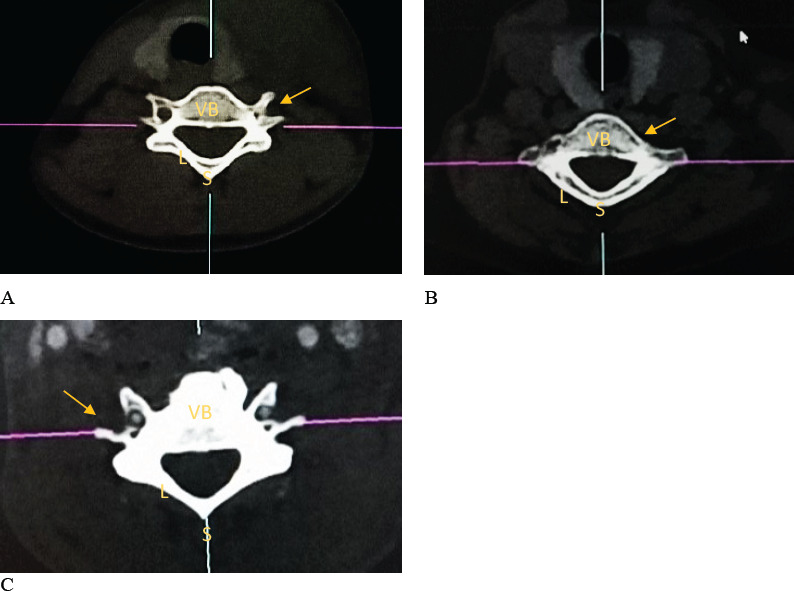
Constricted and Spiculated Transverse Foramina

Of the TF, 42 were constricted (3.19%). Of these, 37 were C7 (88.10%), 2 C6 (4.76%) and 3 C3 vertebrae (7.14%). On the other hand, only 3 C6 TF were spiculated due to presence of incomplete septation. This is illustrated below. (Figure 4).

### Morphometry

The anteroposterior mean for right and left sides combined was 4.60±1.53 mm with a range of 0.30-10.80 mm. The right AP and transverse diameters were significantly greater than those on the left (p < 0.001). The mean AP distance on the right was 4.74±1.44 mm while that on the left was 4.50±1.58 mm. The mean transverse distance on the right was 5.45±1.46 mm while that on the left was 5.28±1.65 mm.

The average right and left AP distance in males was 5.71±1.39 mm and 5.84 ± 1.48 mm respectively, while the average right and left AP distance in females was 5.92 ±1.76 mm and 5.60 ± 1.64 mm. The average right transverse distance in males was 6.20 mm ±1.52 mm, and left transverse distance was 6.23 ±2.30 mm. The average right and left transverse distance in females was 5.98 ±1.45 mm and 5.88 ±1.92 mm respectively. There were no statistically significant sex differences in foraminal diameters (p=268).

A one-way ANOVA was applied and statistically significant level differences were noted in AP and transverse diameters of TF down the cervical spine (p < 0.001). A Tukey Post Hoc multiple comparisons test further established that TF of C1 and those of C7 had the greatest variation in AP and transverse diameters with C3-C5 foramina having the least difference.

## Discussion

Knowledge of the variations of TF is useful in minimizing intraoperative lesions of vertebral arteries during cervical procedures since minor lesions may result in severe hemorrhages. Findings of the present study are also useful in the interpretation of radiographic films as well as in determination of the etiology of vertebrobasilar insufficiency and vascular syndromes.

### Morphology

Ninety-eight point seven eight percent (98.78%) of vertebrae studied had prevalent TF with only 1.22% having absent TF. Findings by Sumalatha and Manasa (2018) in an Indian population have reported unilateral absence in 15.78% and bilateral absence of TF in 2.63% of the 38 C7 vertebrae studied. Taitz et al. (1978) also reported foraminal absence in 0.83% of the 480 vertebrae studied. Sethi et al. (2014) reported an absence of 2% of the 50 vertebrae studied. C7 vertebral level was the most common level where absence was reported. Absence of TF could be due to complete ossification of the roots of the transverse process during embryological development. As a result, this absence could be an indicator of the absence of the VA or a deviation of the foraminal course of the artery.

The most prevalent shape was type 1 (41.32%). This is similar to findings by Molinet Guerra et al. (2017) and Zaw et al. (2021) in Indian and South African populations respectively. However, this is contrary to the study by Rekha and Neginhal, (2013) in the South Indian population that found type 2 as the most prevalent. These variations may be due to differences in ethnicity posed by differences in underlying genetics and environmental factors 19. Differences in shape down the cervical spine may require level-specific standardization of measurements during screwing when performing cervical spine fusion surgeries.

In addition, accessory foramina had a prevalence of 3.64% in this study which is close to that reported by Zibis et al. (2018) in the Indo-European population (4,57%). Unilateral accessory foramina were found to be more common on the right side than the left. Similar findings have been reported by Gupta and Agarwal, (2019). This side difference may be a useful indicator in predicting the variations in the course and structure of the right VA. Moreover, accessory TF were more common in the lower cervical vertebrae (C5, C6 and C7), mostly C6. Similar findings have been reported by Sharma et al. (2010) and Murlimanju et al. (2011). Bilateral duplication of the transverse foramen could indicate bilateral duplication of the VA. Duplications of the transverse foramen are as a result of compartmentalization or due to dual development of the VA. Thus, TF duplication may be of clinical significance in prediction of accessory verteral arteries.

In addition, incomplete foramina were noted in 0.9%. Studies by Sumalatha and Manasa, (2018) in an Indian population report incomplete TF in 6.66%. Incompleteness of TF may be a result of failure of lateral fusion of the two roots of the transverse process. The incomplete foraminal presentation may be of clinical significance as it may predispose the VA to iatrogenic injury during cervical surgeries.

Spiculations and osteophytic encroachments in TF were encountered in this study. Similar observations were made by Taitz et al. (1978) in an Indo-European population. These spiculations contributed to narrowing of TF. Narrowness and constrictions of TF could be an indicator of narrowness of the VA and its predisposition to compression during sudden neck movements 2. Spicules of incomplete septation, as reported, may pierce the neurovascular structures resulting in vascular insufficiency or persisting pain 2.

### Morphometry

Anteroposterior and transverse diameters of the first cervical TF were largest and those of the seventh cervical TF the smallest. Similar findings have been reported in studies by Kimura et al. (1985) in Japan, Zibis et al. (2018) in Indo-Europe, Mehta and Mokhasi, (2021) in India and El-Dwairi et al. (2021) in Jordan. AP diameters decreased from C2 to C3 then increased to C6 atwhich point the VA enters 24. This might imply that there is a decrease in allowance space available for lateral decompression when moving from a cephalic to a caudal direction. This, however, needs to be verified with other bony structures like the uncinate process to determine the laterality of decompression. Surgeons should take note of the varying AP dimensions down the cervical spine and use level-specific techniques during spinal fixation and posterior discectomy and fusion.

In line with findings by Yadav et al. (2014), but contrary to those of Epstein et al 1969 and Taitz et al 1985, transverse diameters of TF were found as being larger on the right side. A study by Sangari et al., (2015), reported no statistically significant side differences. Side differences may be due to population and genetic differences (HOX expression) in the development of the VA and TF. Thus, caution should be taken during pedicle screw insertion especially on the right side in the Kenyan population.

In conclusion, side, sex and level dependent morphologic and morphometric variations of TF were noted in the adult Kenyan population. This is of clinical importance to surgeons to prevent intraoperative damage of vertebral vessels during cervical procedures and to radiologists for image analysis.
